# Cell Models for the Investigation of the Role of the Mucin MUC1 Extracellular Domain in Metastasizing

**Published:** 2014

**Authors:** M. S. Syrkina, M. A. Rubtsov, D. M. Potashnikova, Y. D. Kondratenko, A. A. Dokrunova, V. P. Veiko

**Affiliations:** Department of Molecular Biology, M.V. Lomonosov Moscow State University, Leninskie Gory, 1/12, 119899, Moscow, Russia; Department of Cell Biology and Histology, M.V. Lomonosov Moscow State University, Leninskie Gory, 1/12, 119899, Moscow, Russia; Bioengineering Department, M.V. Lomonosov Moscow State University, Leninskie Gory, 1/12, 119899, Moscow, Russia; A.N. Bach Institute of Biochemistry, Russian Academy of Science, Leninsky prospekt, 33/2, 119071, Moscow, Russia

**Keywords:** mucin, MUC1, cell models, HT-29, cancer, metastasis

## Abstract

The speculations on the role of MUC1, a substance which is overexpressed in glandular cancer cells, on the metastatic potential of such cells are rooted in data that seem to indicate that cell malignization correlates with a change from the apical localization of mucin MUC1 to a peripheral one. Nonetheless, the role of MUC1 in cancer metastasizing remains far from clear. The major hurdle remains the absence of adequate cell models. The aim of the present study was to create cell models that present different fragments of the human mucin MUC1 extracellular domain on their surface. Genetic constructions were generated on the basis of the plasmid vector pEGFP-N3. These constructions contain fusion genes coding for chimeric proteins composed of different combinations of mucin MUC1 functional domains and identification markers (FLAG-epitope, located at the N-terminus, and EGFP, located at the C-terminus of the chimeric proteins). These constructions were used for a stable transformation of HT-29 human cancer cells. The transformants obtained were characterized by flow cytometry. The low expression level of endogenous mucin MUC1 and the high expression level of recombinant proteins were confirmed by real-time PCR. The microscopic examination of the transformed cells confirmed the membrane localization of the fusion proteins. The resulting cell models could be used to investigate the role of the mucin MUC1 domains in cancer cell metastasizing. The obtained cells are used as an applicable model of MUC1-expressing cancers and might be used to study the role of different functional fragments of mucin MUC1 in metastasizing.

## INTRODUCTION

Metastasizing, a process characterized by an increased ability of cells to invade and migrate through the endothelium, is considered to be one of the key processes underlying cancer development. The emergence of the ability to metastasize in tumor cells is usually associated with poor prognosis [1]. The molecular mechanisms of the processes that lead to changes in a cell’s metastatic potential have been the subject of active investigation for several decades. It has been established that the molecules that interact with the extracellular matrix [2, 3] or cell adhesion molecules [4, 5] are involved in these processes. One such molecule is human mucin MUC 1. Experimental data indicating that an increased expression of MUC 1 correlates with a reduced aggregating ability of cells in a culture [6] has prompted researchers to postulate that this glycoprotein magnifies the metastatic potential of tumor cells.

Membrane glycoprotein MUC 1 is normally located on the apical surface of the epithelial cells that line the airways and ducts of glands and performs the functions of moisturizer and lubricant. The expression of the MUC 1 gene increases in malignant, transformed cells [7-9]; cellular localization [10, 11] and the glycosylation pattern of mucin MUC 1 changes [12].

There are several functional domains in the mucin MUC 1 structure: an extracellular, a transmembrane, and a cytoplasmic one. The data on the influence of the different MUC 1 domains on the metastatic potential of tumor cells are rather contradictory [13-15]. Only the role of the cytoplasmic domain has been established rather unequivocally: the signaling pathways this fragment participates in and the intracellular molecules it interacts with have been identified [16-18]. However, the functional role of the MUC 1 extracellular domain in this process is not entirely clear, although it is known that the changes in the mucin molecule in the course of malignant cell transformation basically affect its extracellular region. A change in the glycosylation pattern of the mucin MUC 1 extracellular domain in tumor cells leads to the formation of tumor-specific antigenic epitopes. It is assumed that the glycosylation pattern of mucin can significantly affect the tumor cell invasiveness. Thus, it has been shown that the carbohydrate components of tumor-associated (but not “normal”) mucin MUC 1 are able to bind the selectins produced by activated endothelium cells [19]. In turn, the selectins mediate the interaction with the receptors on the endothelial cell surface, which apparently promotes the attachment of a metastatic cell to the vessel wall and subsequent invasion. Furthermore, metastatic cells are characterized by the presence of certain glycosidic epitopes [20, 21]. The glycoside epitopes syalil Lewisa (sLea) and syalil Lewisx (sLex) have been identified for MUC 1; their presence is associated with the metastasizing ability of cells. In particular, it has been shown that the sLea content gradually increases during the neoplastic transformation of intestinal cells [22]. Increased amounts of dimeric forms of sLex were also found in subpopulations of lung adenocarcinoma cells demonstrating a considerable ability to form colonies in the lungs of athymic mice [23]. These data indirectly attest to the participation of the mucin MUC 1 extracellular domain in the amplification of the metastatic potential of tumor cells in mammals.

Nevertheless, it remains impossible to study the effect of the MUC 1 extracellular domain on tumor cells metastasizing without adequate cell models. Cell models of aggressive forms of MUC 1-expressing cancers can be used to test both anticancer drugs and diagnostic tools aimed at recognizing the extracellular fragment of mucin MUC 1.

Since expression of mucin MUC 1 is typical of most epithelial tumors, the development of such agents could cover a wide range of cancer diseases.

## EXPERIMENTAL

**Materials**

We used salts purchased from Merck (Germany) and salts of domestic production (special purity grade); components for microbiological media (Difco, USA); agarose; enzymes and kits for PCR , RT -PCR , real-time PCR , extraction of DNA fragments from agarose gel and isolation of the plasmid DNA, as well as DNA and RN A markers (Thermo Scientific, USA), TR Izol (Sigma, USA), and DEPK (Sigma, USA).

Cell-culturing was performed using a Dulbecco’s modified Eagle’s medium (DMEM), Versen solution, trypsin solution (PanEco, Russia), fetal calf serum (Hyclone, USA), antibiotics geneticin (G418), penicillin, and streptomycin (Sigma-Aldrich, USA).

**Bacterial strains and cell cultures**

The *Escherichia coli *JM110 strain (e14-[F ‘traD36 pro- AB + lacIq lacZΔM15] hsdR17 (rK-mK +)) came from the collection of the State Research Institute of Genetics and Selection of Industrial Microorganisms (“Genetika”). We used human cell cultures of breast cancer (MCF-7), cervical cancer (HeLa), and colorectal adenocarcinoma (HT-29).

**Synthetic oligonucleotides**

The oligonucleotides used were synthesized by OOO DNK-Sintez; their sequences are listed in Table 1.

**Table 1:** Sequences of oligonucleotides used in this work

Name	5'→3' sequence
FLAG_F	AGCTTGACTACAAGGACGATGACGATAAGA
FLAG_R	AGCTTCTTATCGTCATCGTCCTTGTAGTCA
USTR_F	ACGTCTCGAGATGACACCGGGCACCCAGT
TM_F	TGGGGGATCCGTGCCAGGCTGGGGCAT
TM_F(2)	AAGCTTGTGCCAGGCTGGGGCAT
CT_R	ACGTGGATCCCCAAGTTGGCAGAAGTGGC
TM_R	GCTAGGATCCGCACTGACAGACAGCCAAGGC
USTR_R	TGACAAGCTTCCCCAGGTGGCAGCTGAA
GAPDH_F	CAAGGTCATCCATGACAACTTTG
GAPDH_R	GTCCACCACCCTGTTGCTGTAG

**RNA isolation from cell culture**

The cells washed with DPBS were pelleted by centrifugation (4 min, 1000 rpm) and re-suspended in 1 ml of the TR Izol reagent. 200 µl of chloroform was added; the pellet was stirred and incubated for 2–3 min at room temperature, then centrifuged (15 min, 13,000 rpm, +4°C), and the upper aqueous phase was collected in a clean tube. An equal volume of isopropanol was added; the mixture was incubated for 10 min at room temperature whereupon nucleic acids were precipitated by centrifugation (10 min, 13,000 rpm, +4°C). The pellet was washed with 75% ethanol, dried, and dissolved in water containing 0.1% DEPK. DNA was removed by digestion with DNase I following the manufacturer’s recommendations.

**cDNA synthesis**

cDNA was synthesized by the reverse transcription technique using a RevertAid First Strand cDNA Synthesis Kit (Thermo Scientific, USA) following the manufacturer’s recommendations.

RNA isolated from a MCF-7 cell line and the specific primers USTR _R for cDNA(ustr) and CT _R for cDNA(tmct) were used to obtain cDNA fragments coding for the functional fragments of mucin MUC 1.

RNA isolated from HeLa and HT-29 cells was used to obtain cDNA. cDNA (GAPDH) was obtained using GAPDH_R oligonucleotide. cDNA (MUC 1) was prepared using the oligonucleotide TM_R; cDNA (rMUC 1), using FLAG_R oligonucleotide.

**Preparation of DNA fragments encoding mucin MUC 1 functional fragments**

The oligonucleotides for producing mucin gene fragments were chosen after the cDNA sequence of human mucin MUC 1 had been analyzed [24].

The *Ustr *fragment was obtained by PCR with cDNA(ustr) and the primers USTR _F and USTR _R; the* tmct *fragment was obtained by PCR with cDNA(tmct) and the primers TM_F and CT _R; the *tm *fragment was obtained by PCR with cDNA(tmct) and the primers TM_F (2) and TM_R. The PCR products were separated by electrophoresis in 1% agarose gel and extracted from the gel using a GeneJET Gel Extraction Kit (Thermo Scientific, USA). The resulting fragments were cloned into the vectors pUC 18 – *ustr *at the restriction sites XhoI and HindIII; *tm*, at the restriction sites HindIII and BamHI; and *tmct*, at the restriction site BamHI. The following plasmids were obtained: pUC 18-USTR , pUC 18-TM, and pUC 18-TMCT . Correspondence of the nucleotide sequences of the cloned fragments to the expected ones was confirmed by sequencing.

The *Tr21 *fragment was obtained using the technique developed to produce fragments containing different numbers of tandem repeats from the VNTR region of the human MUC 1 gene [25], and it was cloned into the vector pUC 18 – TR 21 (at the restriction sites HindIII and BamHI).

**Production of expression vectors containing different fragments of the mucin MUC1 gene**

Assembly of the plasmids was performed on the basis of vector pEGFP-N3 (Clontech, USA). The *f *fragment was obtained by annealing the oligonucleotides FLAG_F and FLAG_R.

**Fig. 1:**
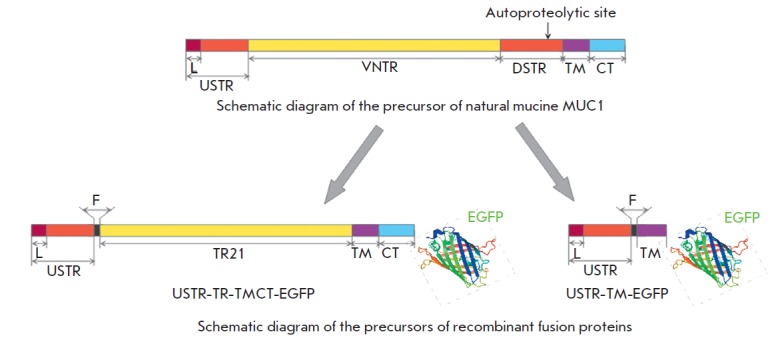
Arrangement of the major functional domains of endogenous mucin MUC1 and recombinant fusion proteins. L – signal peptide; VNTR – region with a variable number of tandem repeats; TR21 – 21 tandem repeat from VNTR; USTR and DSTR – non-regular repeats located upstream of VNTR and downstream of VNTR, respectively; F – FLAG epitope, TM – transmembrane domain, CT – cytoplasmic domain

The vector pUSTR -TR -TMCT -EGFP was obtained by sequential cloning of the fragments *ustr *(at the restriction sites XhoI and HindIII), *tr21 *(at the restriction sites HindIII and BamHI), *tmct *(at the restriction site BamHI), and *f *(at the restriction site HindIII). Vector pUSTR -TM-EGFP was obtained by sequential cloning of the fragments *ustr *(at the restriction sites XhoI and HindIII), *tm *(at the restriction sites HindIII and BamHI), and *f *(at the restriction site HindIII). The desired fragments were obtained by restriction of the vectors pUC 18-USTR , pUC 18-TM, pUC 18-T MCT and pUC 18-TR 21, followed by extraction from the agarose gel. Thus, the pUSTR -TM-EGFP and pUSTR -TR - TMCT -EGFP expression vectors were prepared; they contained genes encoding the USTR -TM-EGFP and USTR -TR -TMCT -EGFP fusion proteins, respectively. A schematic representation of the mutual arrangement of the domains in the structure of the recombinant proteins as compared with the structure of natural mucin is shown in Fig. 1.

**Transfection**

Vector pEGFP-N3 and the plasmids pUSTR -TM-EGFP and pUSTR -TR -TMCT -EGFP were used for transfection. Transfection of HT-29 cells was performed using a Gene Pulser Xcell Total electroporation system (Bio- Rad, USA). 20 µl of plasmid DNA (0.4 µg/µl) and 180 µl of the cell suspension (5×10^6^ cells/ml) were placed in a 2-mm electroporation cuvette; the mixture was stirred and subjected to an electric field (500 V/cm, 10 pulses of 1 ms). The cells were then seeded in culture dishes and grown in a complete DMEM medium for 3 days, washed with DPBS, supplied with fresh DMEM containing antibiotic G418 (700 µg/ml), and cultured for 10–14 days. The most brightly glowing colonies were picked and placed into a 96-well plate. The stably transfected cells were analyzed using a flow cytometer (BD FACS AriaIII, USA).

**Flow cytometry**

For the flow cytometry analysis, the cells were washed with a DPBS solution three times and re-suspended in DPBD to approximately 1×10^6^ cells/ml.

**Fluorescence microscopy**

The cells were seeded on glass coverslips and analyzed after 24 h using a Nikon Eclipse Ts100 fluorescent microscope (Nikon, Japan) or an LSM510 Meta microscope (Carl Zeiss, Germany).

**Real-time PCR**

PCR was performed using the Maxima SYBR Green qPCR Master Mix 2X (Thermo Scientific, USA) containing the SYBR Green intercalating agent. The concentrations of primers in the reaction mixtures were 0.03 mmol/l. Dilutions of the cDNA sample (10, 100, 1000 and 10,000-fold) were used to plot a calibration curve. Reactions without cDNA and without reverse transcriptase M-MuLV were used as a control. All experiments were performed in triplicate.

The expression level of endogenous MUC 1 in nontransfected cells was determined using the MUC 1 cDNA and TM_F/CT _R primer pair; the expression level of recombinant mucin MUC 1 was analyzed using the cDNA rMUC 1 and USTR _F/FLAG_R primers.

The results were normalized to the expression level of the GAPDH gene using cDNA (GAPDH) and GAPDH_F/GAPDH_R primers.

**Determination of the growth pattern of cells expressing recombinant proteins**

The cells of each stable line – HT-29_EGFP, HT- 29_USTR -TM-EGFP and HT-29_USTR -TR -TMCT - EGFP – were seeded in culture dishes 3.5 cm in diameter (500,000 cells per dish). After 3 days of growth, the cells were washed with DPBS and their pattern growth was analyzed under a microscope. The number of cells was determined by counting in a Goryaev’s chamber.

## RESULTS AND DISCUSSION

**Cell line selection**

A number of requirements should be taken into account when choosing a cell line to obtain the desired models.

Since these cell models are supposed to be used to study the properties of a molecule that presumably increases the metastatic potential of cells, a parent cell line demonstrating low initial metastasizing ability should be chosen.

Furthermore, in an ideal case, parent cells should not express endogenous mucin MUC 1 at all to avoid ambiguous results. However, in about 90% of malignancies, there is expression of mucin MUC -1; that is why a tumor cell line with a significantly reduced expression level of endogenous MUC 1 should be chosen.

The glycosylation pattern of mucin is also important when selecting a parent cell line. In 1997, Burdick *et al. *[20] analyzed the glycosylation pattern of recombinant mucin MUC -1 isolated from four different cell lines. According to the results of glycoprotein hybridization with monoclonal antibodies against the most common tumor-associated glycosidic epitopes occurring in natural mucin, it was shown that only recombinant mucin derived from the HT- 29 cell line comprises the sLea and sLex epitopes [20] that are characteristic of cells from aggressive forms of tumors of epithelial origin. According to the published data [2], the HT-29 cell line exhibits a low metastatic potential as well. We compared the expression levels of endogenous mucin in HT-29 and HeLa cells using real-time PCR .

It is known that different isoforms (including the secreted isoform [26]) of mucin MUC -1 can be produced by cells via alternative mRN A splicing. In our case, it was important to estimate the expression level of the membrane-bound forms of this glycoprotein only; therefore, the TM_F and TM_R primers to the cDNA fragment encoding the transmembrane domain of MUC 1 were used in the PCR reaction. Table 2 shows the results of an evaluation of the *MUC1* gene expression level in the HT-29 and HeLa cell lines.

**Table 2:** Real-time PCR data showing the content of mRNA of endogenous mucin MUC1 in HT-29 and HeLa cells

mRN A/cell line	Mean ultimate growth	Mean initial count	% with respect to GAPDH
GAPDH / HeLa	25.42	3214.1	100
GAPDH/ HT-29	24.19	11795.4	100
MUC 1 / HeLa	25.35	4007.57	125
MUC 1 / HT-29	28.24	265.28	2.25

The results of real-time PCR demonstrate that the amount of MUC 1 mRN A in HT-29 cells is about two orders of magnitude lower than that in HeLa cells. This fact is indicative of the low baseline level of *MUC1 *gene expression in HT-29 cells.

Thus, we relied on the published data and our own results and chose the HT-29 cell line to create models of tumor cells that express recombinant proteins comprising certain functional fragments of mucin MUC 1.

**Construction of model structures**

When studying the role of various functional fragments of MUC 1, researchers usually use DNA encoding natural MUC 1 [27-29]. However, natural MUC 1 is a heterodimer, wherein the extracellular N-terminal subunit is linked to the membrane-bound one by means of stable non-covalent interactions. These interactions might be disturbed, resulting in a “discarding” of the N-terminal subunit from the cell surface [30]. Such a dissociation in the course of studying a protein subunit containing the extracellular domain of mucin might distort the anticipated results. That is why we needed to construct genes of recombinant proteins that have no autoproteolytic sites but contain the desired functional fragments.

**Fig. 2:**
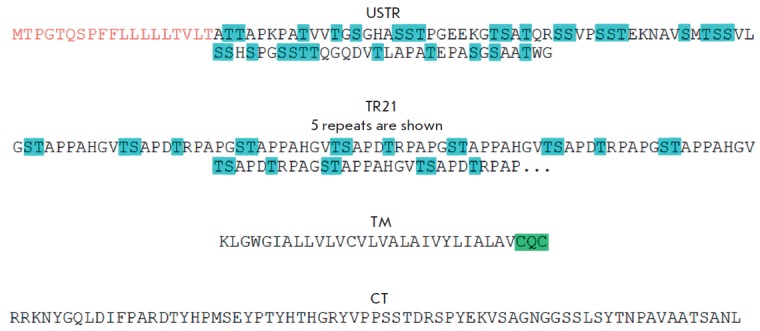
The primary structure of the mucin MUC1 fragments used to create the constructions. The potential O-glycosylation sites are highlighted in blue; the sequence anchoring the protein in the plasma membrane is highlighted in green. The signal peptide sequence is shown in red

The main difference between tumor-associated and normal mucin MUC 1 is that certain glycosidic epitopes are present on the surface of the extracellular domain in the former case: the carbohydrate components of this molecule are supposed to be involved in the interactions with extracellular matrix molecules and, consequently, in metastasizing. Therefore, the recombinant protein to be constructed should contain fragments of the extracellular domain bearing O-glycosylation sites. A large number of the Ser and Thr residues that are supposed to carry oligosaccharides are located in the tandem repeat region (VNTR ), where each repeat contains 20 amino acid residues, including five potential Oglycosylation sites. Following the technique developed to construct fragments that encode different amounts of repeats from VNTR of human mucin MUC 1 [25], a fragment encoding 21 tandem repeats was obtained (*Fig. 2*).

A large number of potential O-glycosylation sites are also characteristic of the areas of degenerate repeats (USTR and DSTR ). Since the DSTR region contains an autoproteolytic site, this fragment was not used during the construction. Meanwhile, a DNA fragment coding for the USTR sequence needs to be obtained (see *Fig. 2*).

Finally, to ensure exposure of the extracellular domain of MUC -1 in the recombinant proteins to the extracellular space, the latter needs to contain at least the transmembrane domain (TM) and the CQC sequence, thus providing membrane localization of the glycopro tein [31]. The amino acid sequence of the transmembrane domain is shown in Fig. 2.

The nucleotide sequence encoding the cytoplasmic domain (CT , see *Fig. 2*) was required to construct a gene of full-length mucin lacking only the region encoding the autoproteolytic site and some degenerate repeats.

The fragments encoding the USTR , TM, and CT of mucin MUC 1 were amplified using cDNA of mucin MUC 1 synthesized on the total mRN A derived from MCF-7 breast cancer cells.

Two expression constructs were obtained using the aforementioned fragments; the constructs contained genes of chimeric proteins fused to EGFP at the C-terminus: full-length MUC 1 (pUSTR -TR -TMCT -EGFP) and that of the lacking tandem repeats and the cytoplasmic domain (pUSTR -TM-EGFP) (*Fig. 1*).

We inserted the sequence encoding FLAG-epitope between the region of tandem repeats and the fragment encoding degenerate repeats to make it possible to identify the N-terminal region of recombinant proteins using specific antibodies.

**Fig. 3:**
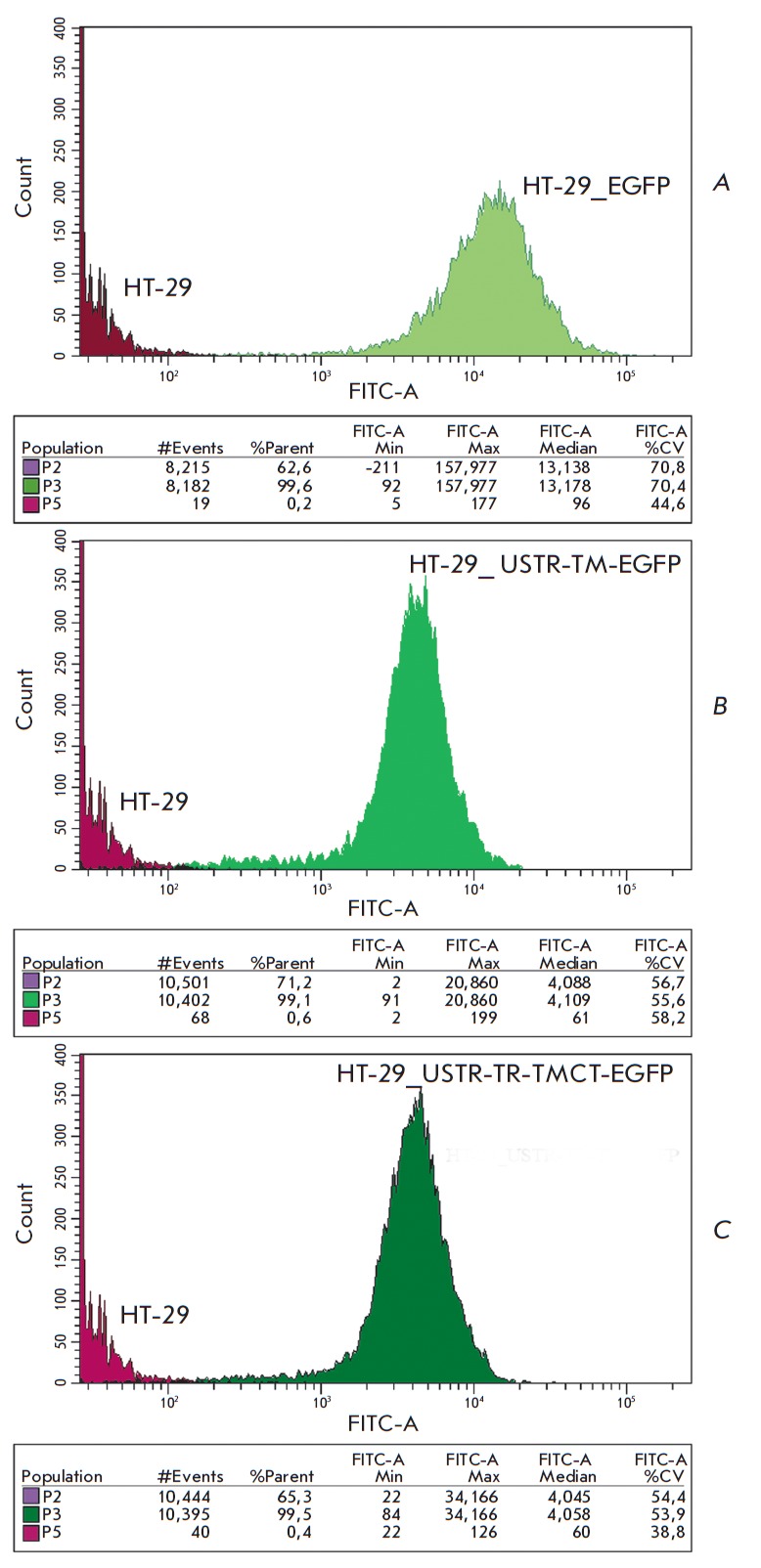
Fluorescence of HT-29 cells stably transfected with pEGFP-N3 (A), pUSTR-TM-EGFP (B), pUSTR-TR-TMCTEGFP (C)

Preparation and characterization of cellular models The expression level of recombinant proteins was assayed by flow cytometry of stable transfected cells by virtue of EGFP fluorescence (*Fig. 3*).

The expression level of recombinant mucin (rMUC 1) in stable transfected cells was analyzed by realtime PCR and compared to the expression level of the housekeeping GAPDH gene. The results of the measurement of the expression level of recombinant protein genes in stable transfected HT-29 cells are shown in Table 3.

**Table 3:** Real-time PCR data showing the content of mRNA of recombinant proteins in stable transfected HT-29 cells

mRN A/cell line	Mean ultimate growth	Mean initial count	% with respect to GAPDH
GAPDH / HT-29_EGFP	14.30	730.65	100
GAPDH / HT-29_USTR-TM-EGFP 1	12.76	1922.36	100
GAPDH / HT-29_USTR-TR-TMCT-EGFP	13.21	1347.97	100
rMUC1 / HT-29_EGFP-N3	22.62	0.94	0.129
rMUC1 / HT-29_USTR-TM-EGFP 1	14.34	373.82	19.446
rMUC1/HT-29_USTR-TR-TMCT-EGFP	14.75	278.03	20.626

It was shown that the mRN A content in the recombinant proteins USTR -TM-EGFP and USTR -TR -TMCT -EGFP in stable transfected HT-29_USTR -TMEGFP and HT-29_USTR -TR -TMCT -EGFP cells is approximately 20% of the content of GAPDH mRN A. If we take into account that the mRN A content of endogenous mucin MUC 1 in the HT-29 cell line is 2.25% (Table 2), a conclusion can be drawn that the mRN A of recombinant mucin in stable transfected HT-29 cells is approximately 85% of all the MUC 1 mRN A (both endogenous and recombinant).

**Fig. 4:**
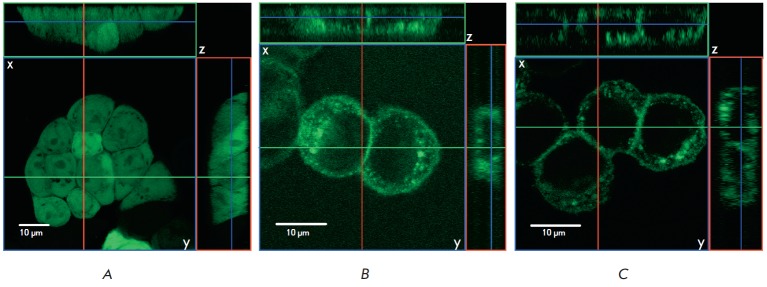
EGFP fluorescence localization in the stable transfected HT-29 cells. A – HT-29_EGFP, B – HT-29_USTR-TMEGFP, C – HT-29_USTR-TR-TMCT-EGFP. The upper and side panels of each image demonstrate an optical section of the 3D image in the xz and yz planes, respectively

An analysis of the localization of recombinant proteins by fluorescence microscopy has shown that the signal in HT-29_EGFP cells in the green part of the spectrum localizes both in the cytoplasm and in the nucleus (*Fig. 4A*), while in HT-29_USTR -TM-EGFP and HT-29_USTR - TR -TMCT -EGFP cells, the signal predominantly localizes in the plasma membrane (*Fig. 4B, C*).

An analysis of the 3D confocal microscopy images demonstrated that proteins containing fragments of mucin fused with EGFP predominantly have a peripheral membrane localization in contrast to the EGFP protein, which was expectedly detected in the cytoplasm and inside the nucleus (*Fig. 4*, top and side panels of each image).

**Fig. 5:**
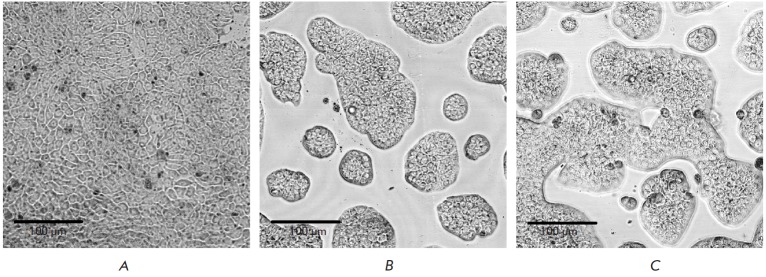
Effect of the expression of recombinant proteins on the growth pattern of stable, transfected HT-29 cells in culture. A – EGFP-expressing cells, B – USTR-TM-EGFP-expressing cells, C – USTR-TR-TMCT-EGFP-expressing cells.

Finally, we found that the resulting cell lines present some differences in their growth patterns. While growing HT-29_EGFP cells form a monolayer (*Fig. 5A*), HT-29_USTR -TM-EGFP and HT-29_USTR -TR -TMCT -EGFP cells tend to form individual “islands” (*Fig. 5B, C*). If we take into account the fact that equal amounts of cells (500,000) were seeded in these experiments and that their number also differed insignificantly after 3 days of growth, the assumption about the difference in cell division intensity needs to be rejected. In our opinion, the observed pattern is indicative of the differences in the adhesion properties of the obtained cell lines caused by the presence of recombinant proteins containing mucin 1 MUC fragments on their surface.

## CONCLUSIONS

We used HT-29 cell lines to obtain cell models expressing proteins fused with EGFP and constituting fragments of the extracellular domain of human mucin MUC 1 bearing O-glycosylation sites on the outer cell surface. The main difference between the resulting models was either the presence or absence of a domain containing 21 tandem repeats in the structure of recombinant proteins. The models obtained are characterized by a high expression level of recombinant proteins and low expression level of endogenous mucin MUC 1. The model cell lines have different growth patterns compared to EGFP-expressing cells.

## Abbreviations

PCR: polymerase chain reaction
RT-PCR: polymerase chain reaction with reverse transcription;
PBS: phosphate buffered saline
DPBS: Dulbecco’s phosphate buffered saline
BSA: bovine serum albumin
GAPDH: glyceraldehyde-3-phosphate dehydrogenase
